# Effectiveness of an interactive music tablet intervention to reduce procedural anxiety in children: a randomized controlled trial

**DOI:** 10.1038/s41598-026-66888-1

**Published:** 2026-08-24

**Authors:** Roland Imle, Ann-Sophie Wiemer, Katharina Hartwig, Christiane Hillebrenner, Stephan Vedder, Sven F. Garbade, Christina Blume, Estelle Willuth, Thomas Mueller, Mira Boos, Julian Koenig, Dorothee von Moreau, Patrick Guenther, Philipp Romero

**Affiliations:** 1 Division of Pediatric Surgery, Department of General, Visceral and Transplantation Surgery, University Hospital Heidelberg, 69120 Heidelberg, Germany; 2 Department of Pediatric Oncology, Hematology and Immunology, Heidelberg University Hospital, Heidelberg, Germany; 3 Hopp Children’s Cancer Center Heidelberg (KiTZ), University Children’s Hospital Heidelberg, Im Neuenheimer Feld 430, 69120 Heidelberg, Germany; 4 National Center for Tumor Diseases (NCT), NCT Heidelberg, a partnership between DKFZ and Heidelberg University Hospital, Heidelberg, Germany; 5 Soft-Tissue Sarcoma Research Group, Hopp Children’s Cancer Center, Heidelberg (KiTZ), German Cancer Research Center (DKFZ), 69120 Heidelberg, Germany; 6 Division of Pediatric Neurooncology, German Cancer Consortium (DKTK), German Cancer Research Center (DKFZ), Heidelberg, Germany; 7 mbits Imaging, 69115 Heidelberg, Germany; 8 Department of Pediatrics I, Division of Pediatric Neurology and Metabolic Medicine, Heidelberg University, Medical Faculty of Heidelberg, 69120 Heidelberg, Germany; 9 Department of Neuropathology, German Cancer Research Center (DKFZ), University Hospital Heidelberg and CCU Neuropathology, German Consortium for Translational Cancer Research (DKTK), Heidelberg, Germany; 10 Department of Anesthesiology, University Hospital Heidelberg, 69120 Heidelberg, Germany; 11 Faculty of Medicine and University Hospital Cologne, Department of Child and Adolescent Psychiatry, Psychosomatics and Psychotherapy, University of Cologne, Cologne, Germany; 12 Institute of Music Therapy, Hamburg University of Music and Drama, 20148 Hamburg, Germany

**Keywords:** Diseases, Health care, Medical research

## Abstract

Procedural anxiety in children is common and associated with maladaptive behavior, reduced cooperation, and increased perioperative stress. Although music-based interventions show promise, few are designed for routine clinical care. We evaluated an interactive tablet-based music intervention (“MuMa”) for reducing procedural anxiety in children aged 3–11 undergoing elective surgery. We conducted a single-center, parallel-group, randomized controlled trial including 80 children undergoing elective surgery and their caregivers. Participants were randomized to receive the MuMa intervention preoperatively or standard care. Primary outcomes were child (modified Yale Preoperative Anxiety Scale) and caregiver anxiety (State-Trait Anxiety Inventory), assessed before and after the intervention. Secondary outcomes included heart rate variability, compliance during anesthesia induction, postoperative agitation, and behavioral changes at 1, 4, and 12 weeks post-procedure. Trial registration: German Clinical Trials Registry (DRKS00027873), registered 13 May 2022. Here we show that the intervention reduces preoperative anxiety by 38% (standardized mean difference -0.8; 95% CI -1.2 to -0.3) and integrates readily into routine perioperative workflows. Effects are greatest in children with high baseline anxiety (SMD -1.7; 95% CI -2.8 to -0.5) and in those aged 3–6 years (SMD -0.9; 95% CI -1.5 to -0.3). No clinically relevant differences are observed for secondary outcomes. This interactive music intervention significantly reduces procedural anxiety in children, particularly among younger participants and those with high baseline anxiety. Its low resource requirements and ease of implementation support its potential as a scalable non-pharmacological strategy in pediatric perioperative care. Further research should evaluate its generalizability across diverse settings.

**Supplementary Information:**

The online version contains supplementary material available at 10.1038/s41598-026-66888-1.

## Introduction

For children and adolescents, even minor medical procedures – particularly in the context of surgery and anesthesia induction – often pose an ambiguous threat that is accompanied by considerable psychological distress and anxiety^1,2^. In the perioperative setting, encompassing preoperative holding, transfer to the operating room, and anesthesia induction, 50–80% of children experience significant periinterventional anxiety^3^. High procedural anxiety is not only linked to increased pain perception and non-compliance, but can also delay recovery and contribute to the development of secondary maladaptive behaviors^4^. High anxiety levels are also associated with increased risks of emergence delirium, and heightened anxious behavior during subsequent medical encounters^5,6^. Children under the age of seven, those with insecure attachment styles, and those with anxious parents are particularly at risk^7^.

In addition to commonly used anxiolytic premedication, such as benzodiazepines like midazolam, several non-pharmacological approaches have been proposed to mitigate procedural anxiety^8^. These include creating a child-friendly environment, providing explanations, validating feelings, using age-appropriate distractions, allowing parental presence until the child falls asleep, offering comfort, praise, and attention, and employing anxiolytic interventions like games, virtual reality, and music^9^. However, the consistent use of these strategies remains limited in routine clinical practice, partly due to time constraints, staff resources, and the lack of robust, scalable approaches that reliably reduce anxiety.

For adults, strong evidence supports the effectiveness of non-pharmacological anxiolytic interventions, especially music^10,11^. While positive effects have been reported in children, the pediatric evidence base remains limited. Systematic reviews including pediatric surgical populations^12,13^ and several trials in children undergoing surgery^14–18^ suggest benefit but are constrained by limitations in study design, low statistical power, and a lack of comprehensive anxiolytic strategies, while evidence from non-surgical settings^19^ may not generalize to perioperative care. Many pediatric music interventions rely on simplistic single-song designs^18^ or one-time sessions with music therapists, which are not scalable for routine clinical practice^20,21^.

To address these gaps, we developed an interactive, age-appropriate audiovisual music intervention based on musicological principles, delivered via a tablet/smartphone application, specifically designed for use by children and caregivers. Here, we evaluate the feasibility and effectiveness of this intervention in reducing procedural anxiety in children undergoing elective surgery in routine clinical practice and test the hypothesis that its anxiolytic effects are greatest in children with high baseline anxiety.

## Methods

### Study design and ethical standards

This monocentric, open-label, superiority, parallel-group randomized controlled trial allocated participants 1:1 to an interactive music intervention or standard care and was conducted at the University Children’s Hospital Heidelberg, a tertiary pediatric referral center in Germany. The study was approved by the Ethics Committee of the Medical Faculty of the University of Heidelberg (number S-468/2020). Written informed consent was obtained from all participants and their caregivers. Recruitment occurred from December 2020 to May 2022 (follow-up concluding August 2022), coinciding with the COVID-19 pandemic. The original recruitment period was planned for 1 year, but was extended to 1.5 years because of COVID-19-related constraints. Recruitment was closed after this extended study period, resulting in 80 enrolled participants; the trial was not stopped early for safety or efficacy reasons. The study was registered with the German Clinical Trials Registry (DRKS00027873; UTN U1111-1278-1784) after initiation of recruitment (registered 13 May 2022). The original study protocol (German language version) and statistical analysis plan are provided in the Supplementary Information. The study protocol, eligibility criteria, outcome measures and statistical analysis plan were defined prior to data acquisition and analysis. One ethics-approved protocol amendment (17 February 2021) expanded the eligible population to include additional elective pediatric medical interventions beyond surgery; however, no participants were recruited under this amendment, and all enrolled participants underwent elective surgical procedures under the original protocol. No changes were made to the predefined outcomes, study procedures, or statistical analysis plan after trial commencement. The study followed the CONSORT 2025 statement and its extension for non-pharmacological treatments^22^ and complied with the Declaration of Helsinki.

### Patient and public involvement

Children and caregivers provided informal feedback during the development of the intervention. However, they were not involved in the design, conduct, analysis, interpretation, or reporting of the trial, and no patient or public representatives participated in study oversight or governance.

### Participants

Eligible participants were children aged 3–11 years, scheduled for elective surgery along with accompanying parent or caregiver. Written informed consent was obtained. Exclusion criteria included lack of proficiency in the German language, severe cognitive or audiovisual impairments, and the need for emergency surgery. Participants were assigned 1:1 using a computer-generated randomization sequence prepared by the study statistician in Microsoft Excel 16.0 for 200 planned participants. No stratification or blocking was applied. Allocation assignments were placed in sequentially numbered, sealed, opaque envelopes and opened by investigator AW only after enrollment, immediately before intervention delivery. Investigator AW was responsible for participant enrollment and intervention allocation. The randomization sequence remained concealed from investigator AW until participant enrollment completed and the envelope was opened.

### Blinding

Due to the overt nature of the music intervention, participants and primary outcome assessor AW were not blinded. Secondary outcome assessors (anesthesiologists, recovery room nurses) as well as surgeons, and ward nursing staff were not formally blinded but were unaware of group allocation when the MuMa tablet was not visible, which was the case in the majority of instances, helping to minimize potential bias.

### Intervention

**Fig. 1:**
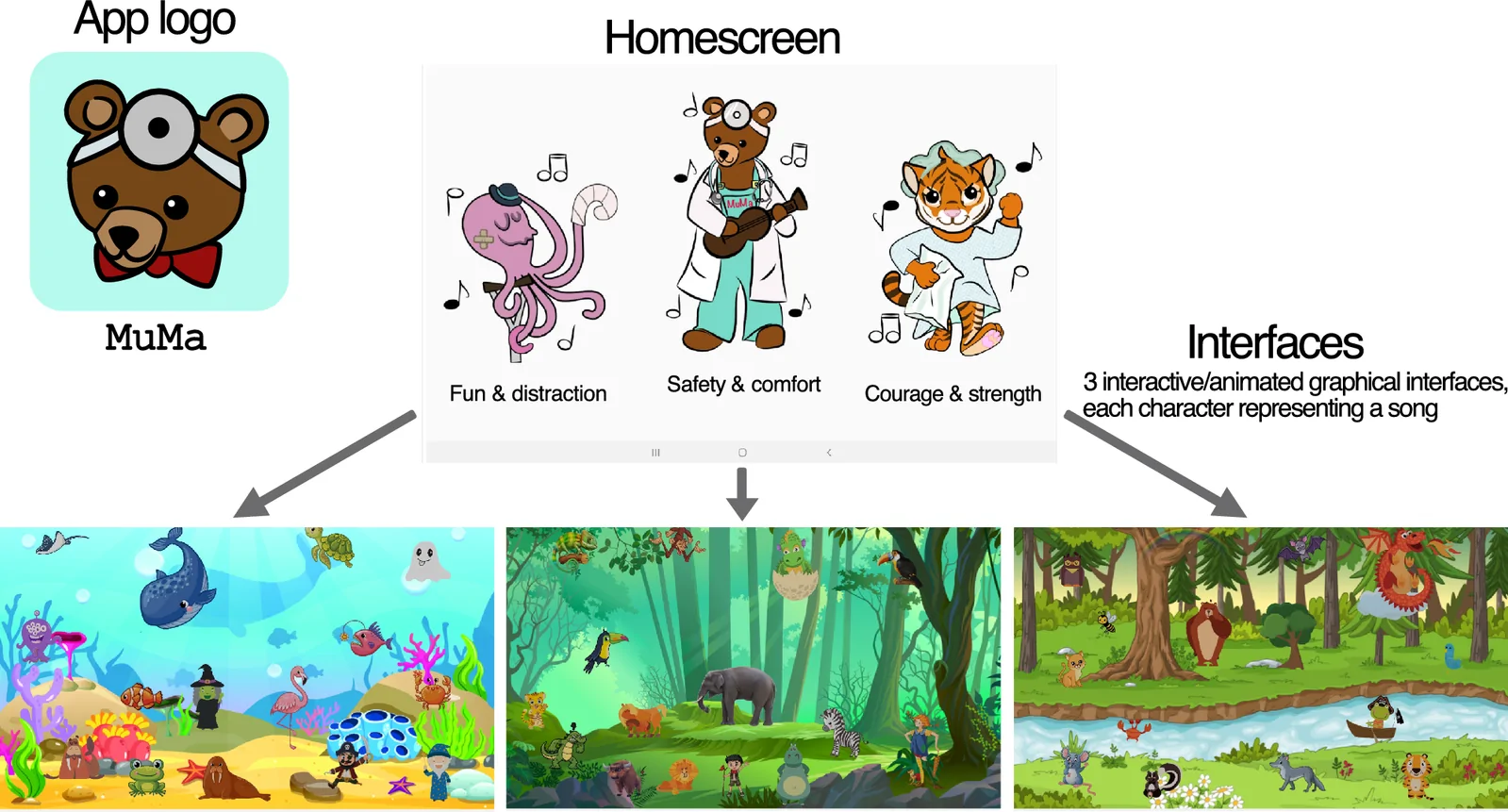
Anxiolytic tablet application design. Screenshot examples of the application logo, home screen, and the three music-based coping categories (fun and distraction, safety and comfort, and courage and strength) integrated into the MuMa application. The application interface was designed by the authors using a combination of original graphical elements and adapted Shutterstock-licensed graphics.

Subjects were randomized into control and intervention groups. Both received identical standard care, including premedication with oral midazolam, a child-friendly environment, and caregiver presence until narcosis induction. When administered, oral midazolam was given after the post-intervention mYPAS assessment and before anesthesia induction; thus, the primary outcome was assessed before pharmacological anxiolysis. The intervention group additionally received a 10.1-inch Samsung Galaxy Tablet A T515, preloaded with the MuMa anxiolytic app version 1.1. MuMa is an interactive, music therapy-informed digital intervention designed to support child-centered coping during the preoperative waiting period. The application presents three coping-oriented music categories – (i) fun and distraction, (ii) safety and comfort, and (iii) courage and strength – through age-appropriate graphical interfaces (Fig. 1). Each category contains a curated set of songs (3 × 15 total) associated with specific emotional and behavioral coping strategies. Children were able to actively select, switch, and replay songs according to their preferences and emotional needs, allowing self-directed engagement rather than passive listening. The audiovisual design combines music with simple visual elements and symbolic characters to facilitate intuitive navigation and emotional identification. Caregivers were present during use and could accompany the child, supporting co-regulation. The intervention lasted at least 10 min during the preoperative waiting period. Intervention delivery was integrated into routine clinical workflow and did not require additional specialized personnel. Intervention adherence was documented for all participants and evaluated based on observed intervention exposure. Tablets were disinfected before and after each use. Additional details regarding intervention development and song selection are provided in the Supplementary Information.

### Outcomes

Primary outcomes included child and caregiver state anxiety before and after the intervention, assessed via the modified Yale Preoperative Anxiety Scale (mYPAS)^23,24^ (observational total score ranging from 23.33 to 100, with higher scores indicating greater anxiety) and the State-Trait Anxiety Inventory – Short Form Y6 (STAI-Y6)^25,26^ (self-reported Likert-scale total score ranging from 6 to 24, with higher scores indicating greater anxiety), respectively. Secondary outcomes included child compliance at narcosis induction (Induction Compliance Checklist, ICC)^27,28^ (observational score ranging from 0 to 10, with higher scores indicating poorer compliance), post-surgery agitation (Excitement Score, ES)^29^ (3-point observational scale: none, marked, substantial agitation), and maladaptive behavioral changes (Post-Hospitalization Behavioral Questionnaire – Ambulatory Surgery, PHBQ-AS)^30,31^ (parent-reported mean Likert-scale score ranging from 1 to 5, with scores above 3 indicating more maladaptive behavior and scores below 3 indicating improved behavior) at 1 week, 1 month, and 3 months post intervention. Heart rate (HR) and its variability (HRV) were measured as a somatic anxiety parameters, but were compromised by movement artifacts (See ECG data processing section below). General fearfulness was assessed on the day of informed consent, 1–4 weeks prior to surgery, with mYPAS for children and the State-Trait Anxiety Inventory – Version YT (STAI-YT)^32,33^ (self-reported trait anxiety score ranging from 20 to 80; higher scores indicate greater trait anxiety, categorized as low [20–39], moderate [40–59], and high [60–80]) for adults. Feasibility was assessed pragmatically across four predefined domains: (i) deliverability within the routine preoperative workflow, (ii) participant engagement with the intervention, (iii) personnel requirements for implementation, and (iv) safety, assessed by monitoring intervention-related adverse events (e.g. discontinuation due to distress, or other unintended negative reactions attributable to the intervention) throughout the intervention period. Participant engagement was evaluated based on documented intervention exposure and classified as high or low. Additional details regarding the applied outcome measures are provided in the Supplementary Information.

### Procedures 

The investigator AW explained the study procedure to parents/caregivers on the day of informed consent, assessing baseline anxiety (mYPAS, STAI-YT) and characteristics such as gender, age, and procedure type (Suppl. Figure 1). AW accompanied the child and caregiver to the waiting area and operating theater and applied the ECG sensor (EcgMove 3 sensor; Movisens GmbH; Karlsruhe, Germany), prior to the intervention. Both groups received standard care and were free to manage their waiting time as they chose. The intervention group was offered the MuMa music intervention via a tablet, with voluntary use pre- and postoperatively, allowing participants to start, switch, or stop the application as desired. Anxiety levels of both parents and children were recorded before and after approximately 30 min of initiating the use the intervention in the preoperative setting, compared to standard care. Child anxiety (mYPAS) was assessed in vivo by the same investigator (AW) before and after the intervention, thereby avoiding inter-observer variability. Prior to study initiation, the investigator received training in mYPAS administration and scoring using the instrument manual and example cases. The observation period between assessments was approximately 30 min and did not differ significantly between groups (Suppl. Figure 2C). The post-intervention mYPAS assessment was completed before administration of preoperative midazolam. Intervention exposure (intensity of MuMa use) was documented and pragmatically classified as high or low based on structured qualitative observations by the study investigator (AW) and caregiver reports, using predefined criteria (active engagement, song selection/switching, sustained attention, and completion of the intended exposure duration). Additional occupation during the waiting period (e.g., use of other distraction strategies) was recorded as a binary variable (yes/no). Additional activities were prospectively categorized as use of personal electronic devices (e.g., smartphones or tablets), reading, toys, drawing, caregiver interaction, or no additional distraction strategy. The ICC was assessed during anesthesia induction by the anesthesiologist, and the ES was recorded upon recovery room arrival by nursing staff. Caregivers were present until the start of narcosis induction and were reunited with their child upon recovery room arrival. PHBQ-AS questionnaires were sent to caregivers for completion via mail and email.

### Anesthesia treatment

All patients received a standardized anesthesia protocol. Following the music intervention, and approximately 30 min before being called to the operating room, most patients received oral premedication with 0.5 mg/kg of midazolam (maximum 8 mg). For venous access, a lidocaine-prilocaine topical anesthetic patch was applied. In the operating room, venous access was established, and anesthesia induction was performed intravenously with Propofol (2–4 mg/kg) or, alternatively, with an inhaled Sevoflurane-oxygen-air mixture. A regional block with 0.2% Ropivacaine was administered as needed, followed by anesthesia maintenance with Sevoflurane-oxygen-air mixture (0.5–1.0 minimum alveolar concentration/MAC). Analgesia was provided with intravenous alfentanil (10 µg/kg), with an additional 10 µg/kg if blood pressure or heart rate increased by ≥ 20% at skin incision. Postoperatively, analgesia included ibuprofen (10 mg/kg), metamizole (15 mg/kg), and/or paracetamol (20 mg/kg), with Piritramid (100 µg/kg) for additional analgesia if needed. Granisetron (10 µg/kg) was used for nausea/vomiting as required. One patient received clonidine (1 µg/kg) for significant postoperative agitation.

### Surgical treatment

The surgical spectrum encompassed a range of typical elective procedures in pediatric surgery, spanning multiple specialties. These included general surgery (i.e., inguinal hernia repair, hydrocele repair, and orchidopexy), traumatology and orthopedics (metal removal), urogenital surgery (hypospadia repair, circumcision, and orchiectomy), and vascular surgery (Hickman catheter implantation and explantation).

### ECG data processing

Electrocardiographic recordings were obtained continuously at 1024 Hz throughout the study period. Raw ECG data were visually inspected and processed using Kubios HRV Premium (Version 3.0)^34^. Recordings with excessive signal distortion or physiologically implausible inter-beat intervals were excluded according to predefined quality criteria. Mean heart rate (HR) and the root mean square of successive differences (RMSSD), a measure of vagally mediated heart rate variability (HRV), were derived from predefined 5-min segments during the intervention period. Detailed preprocessing procedures are provided in Supplementary Information.

### Statistics and reproducibility

Data were collected using a standardized digital form for consistent recording of clinical variables. Analysis was performed using R version 4.4.1 and packages ‘ggplot’, ‘stats’, ‘ggsignif’, ‘ComplexHeatmap’ for data visualization and statistical analysis. Figures were prepared using R version 4.4.1, Affinity Designer version 2.5.5 and Biorender. Data distribution of continuous variables was visualized by histograms and tested for normality with the Shapiro–Wilk test. Non-normally distributed continuous variables were displayed with boxplots, including individual data points and means depicted as black dots and printed out. Data points were jittered slightly, where necessary, to ensure visibility of all data points. Normally distributed continuous variables were summarized as mean ± min/max. Categorical variables were summarized as percentages. All variables were also shown as a heatmap to identify patterns and correlations. Non-normally distributed data were compared between control and intervention groups using Mann–Whitney U tests in one- (Anxiety change, ICC, ES, PHBQ-AS) or two-sided (Baseline anxiety) form depending on hypothesis. The primary outcomes (change in mYPAS score and STAI for caregivers) were compared between intervention and control groups using a Mann–Whitney U test because data were not normally distributed. Normally distributed continuous variables were compared using two-sided Student’s t-tests. Categorical variables were compared using Fisher's Exact tests. Pearson correlation was calculated using the cor function in R. Statistical significance was set to p ≤ 0.05, and effect size was determined using Cohen’s d formula to calculate the standardized mean difference and 95% confidence interval (CI)^35^. For analyses of HRV, mixed-linear effects models were utilized, using the subject ID as random factor, addressing the main effects of segment (over time) and group as well as their interaction. Predefined subgroup analyses were performed according to baseline anxiety level (upper 20% vs. lower 80%) and age group (3–6 vs. 7–11 years). To assess the robustness of the primary outcome against baseline age imbalance, an additional post hoc sensitivity analysis was performed using a linear mixed-effects model with mYPAS as the dependent variable, participant ID as a random effect, and time (pre/post), group (control/intervention), age category (3–6 vs. 7–11 years), and all corresponding interaction terms as fixed effects. A saturated model was fitted and subsequently subjected to bidirectional model selection based on the Akaike Information Criterion (AIC). Linear mixed-effects models were computed using the R package nlme (version 3.1–169). Each participant represented one independent biological unit and contributed one set of outcome measurements. Analyses were performed using all available observations for each outcome measure. Participants were analyzed according to their randomized group assignment. For outcomes with missing data, analyses were based on all available observations without imputation.

### Sample size

Based on previous studies, we estimated high anxiety levels in about 15% of children^36^. Hypothesizing that the anxiolytic intervention might only show relevant anxiolytic effects in high-anxiety children, we calculated a required sample size of n = 100 per group to detect medium effect sizes (Cohen’s d = 0.5) with 80% power at a two-sided 5% significance level using a variance analysis procedure. Logistical constraints and reduced enrollment due to the COVID-19 pandemic limited the study to 80 participants over a 17-month period. No interim analyses or formal stopping guidelines were prespecified or conducted. The sample size for each analysis, including predefined subgroup analyses, is reported in the corresponding Results sections and figure legends.

## Results

**Fig. 2:**
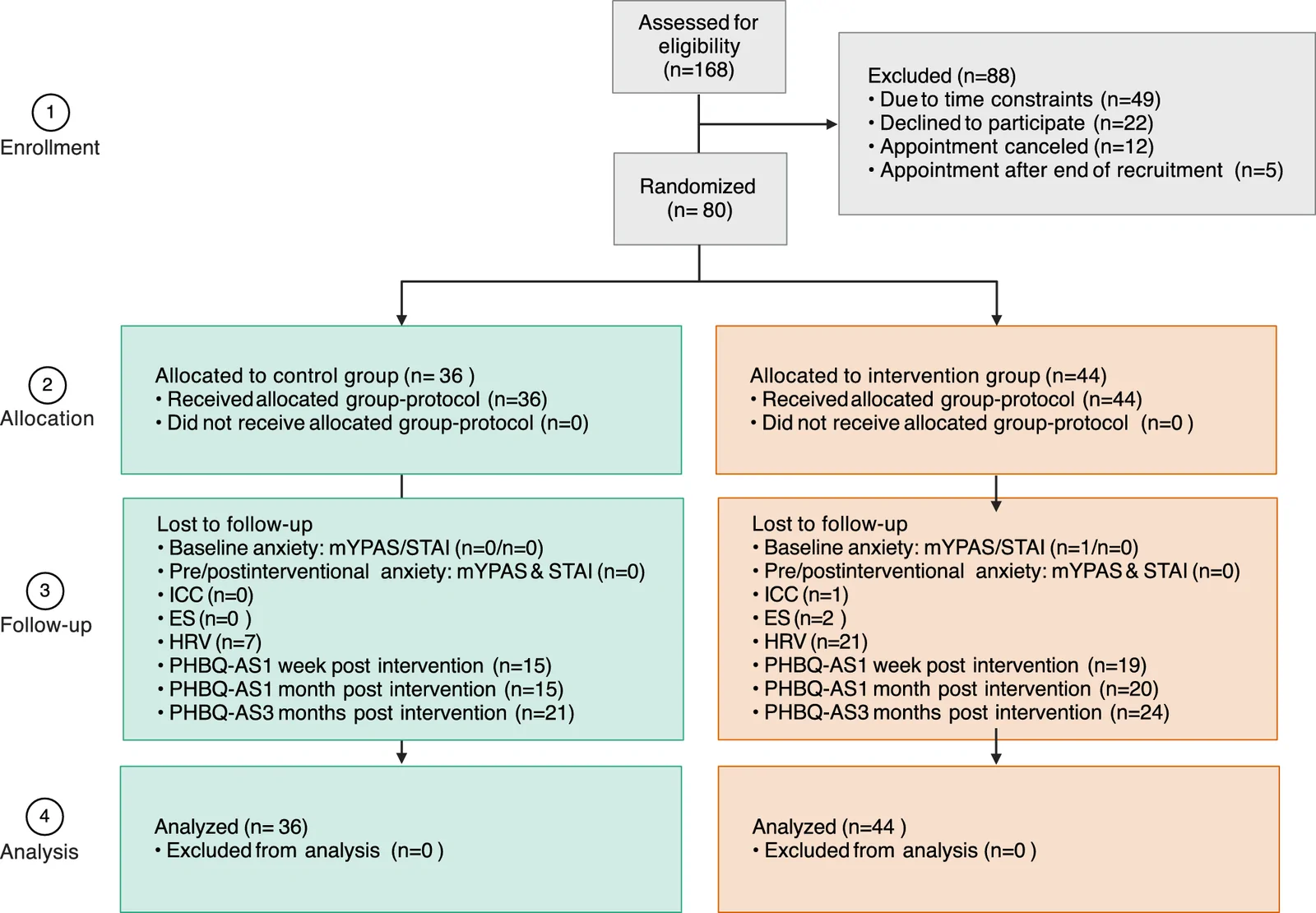
Study flow diagram. Eighty child-caregiver pairs (n = 80) were randomized. The control group received standard care without music intervention.

A total of n = 168 patients were assessed for eligibility, of whom n = 80 were randomized into the control and intervention groups (Fig. 2). Reasons for exclusion and loss to follow-up are summarized in Fig. 2. All randomized participants received the allocated intervention and completed the primary outcome assessment. Missing data were minimal for ICC (n = 1) and ES (n = 2), moderate for HR/HRV because of electrode acceptance issues (n = 17), and highest for post-discharge PHBQ-AS follow-up assessments (41–58%). An additional five ECG recordings were excluded from HR/HRV analyses because of excessive movement artifacts or insufficient signal quality.

Study participants were predominantly non-chronically ill (89%) children, most of whom were treated on an outpatient basis (81%). Boys were slightly more frequent than girls (60% vs. 40%). The mean age was 6.0 ± 2.3 years, with the intervention group slightly younger than the control group (5.5 ± 2.1 vs. 6.7 ± 2.3 years, p = 0.02). Apart from age, no significant differences were observed between the two groups (Table 1). Baseline anxiety, assessed on the day of informed consent, exhibited substantial variance in both children and caregivers, with no significant differences between the intervention and control groups. Mean child mYPAS scores were 37.2 ± 11.4 (control) and 35.8 ± 10.2 (intervention; p = 0.57), indicating clinically relevant anxiety (> 30)^37^(Suppl. Figure 2A), while caregiver trait anxiety (STAI-YT) was low in both groups (32.9 ± 6.8 vs. 33.6 ± 6.7; p = 0.67), as defined by STAI-YT scores of 20–39^38^(Suppl. Figure 2B). The time between anxiety assessments on the day of the intervention was comparable (32.3 ± 12.3 vs. 34.4 ± 11.1 min; p = 0.44) (Suppl. Figure 2C).

**Table 1:** Baseline cohort characteristics.

Summary statistics for child and caregiver data Mean ± SD (min–max) or no./total no. (%)				
Characteristic	All	Standard care group	Intervention group	P value
Patient characteristics				
Age	6.0 ± 2.3 (3.2–10.8)	6.7 ± 2.3 (3.2–10.8)	5.5 ± 2.1 (3.2–10.5)	0.02
Sex				
Male	48/80 (60%)	21/36 (58.3%)	27/44 (61.4%)	0.82
Female	32/80 (40%)	15/36 (41.7%)	17/44 (38.6%)	
Chronic disease	9/80 (11.2%)	5/36 (13.9%)	4/44 (9.1%)	0.72
Number of previous operations	0.9 ± 1.2 (0–7)	1.1 ± 1.0 (0–4)	0.7 ± 1.2 (0–7)	0.17
Location of care				
Inpatient	15/80 (18.8%)	5/36 (13.9%)	10/44 (22.7%)	0.39
Outpatient	65/80 (81.2%)	31/36 (86.1%)	34/44 (77.3%)	
Type of surgery				
Orthopedic	34/80 (42.5%)	16/36 (44.4%)	18/44 (40.9%)	0.95
General surgery	23/80 (28.7%)	10/36 (27.8%)	13/44 (29.5%)	
Urogenital	17/80 (21.2%)	7/36 (19.4%)	10/44 (22.7%)	
Vascular	5/80 (6.2%)	3/36 (8.3%)	2/44 (4.5%)	
Caregiver characteristics				
Age caregiver	37.9 ± 6.1 (23–51)	37.2 ± 6.0 (23–47)	38.4 ± 6.2 (25–51)	0.37
Sex caregiver				
Male	23/80 (28.7%)	9/36 (25%)	14/44 (31.8%)	0.62
Female	57/80 (71.2%)	27/36 (75%)	30/44 (68.2%)	

Values are presented as mean ± standard deviation (range) or n (%). P values were calculated using two-sided Student’s t-tests for continuous variables and Fisher’s exact tests for categorical variables.

### Feasibility

The intervention was successfully delivered within the routine preoperative workflow in all children allocated to the intervention group. Among participants with available data, intervention engagement was high in 38/43 children (88%) and low in 5/43 children (12%) (Suppl. Figure 3). No additional specialized personnel were required for implementation, and no intervention-related adverse events were observed. Additional distraction strategies were commonly used during the preoperative waiting period and were available to participants in both groups. These included personal electronic devices, reading, toys, drawing, and caregiver interaction (Suppl. Figure 3). Among children allocated to the intervention group, 30/44 (68%) combined MuMa with at least one additional distraction strategy, whereas 14/44 (32%) used MuMa as the sole distraction modality.

### The effect of MuMa on preoperative anxiety

**Fig. 3:**
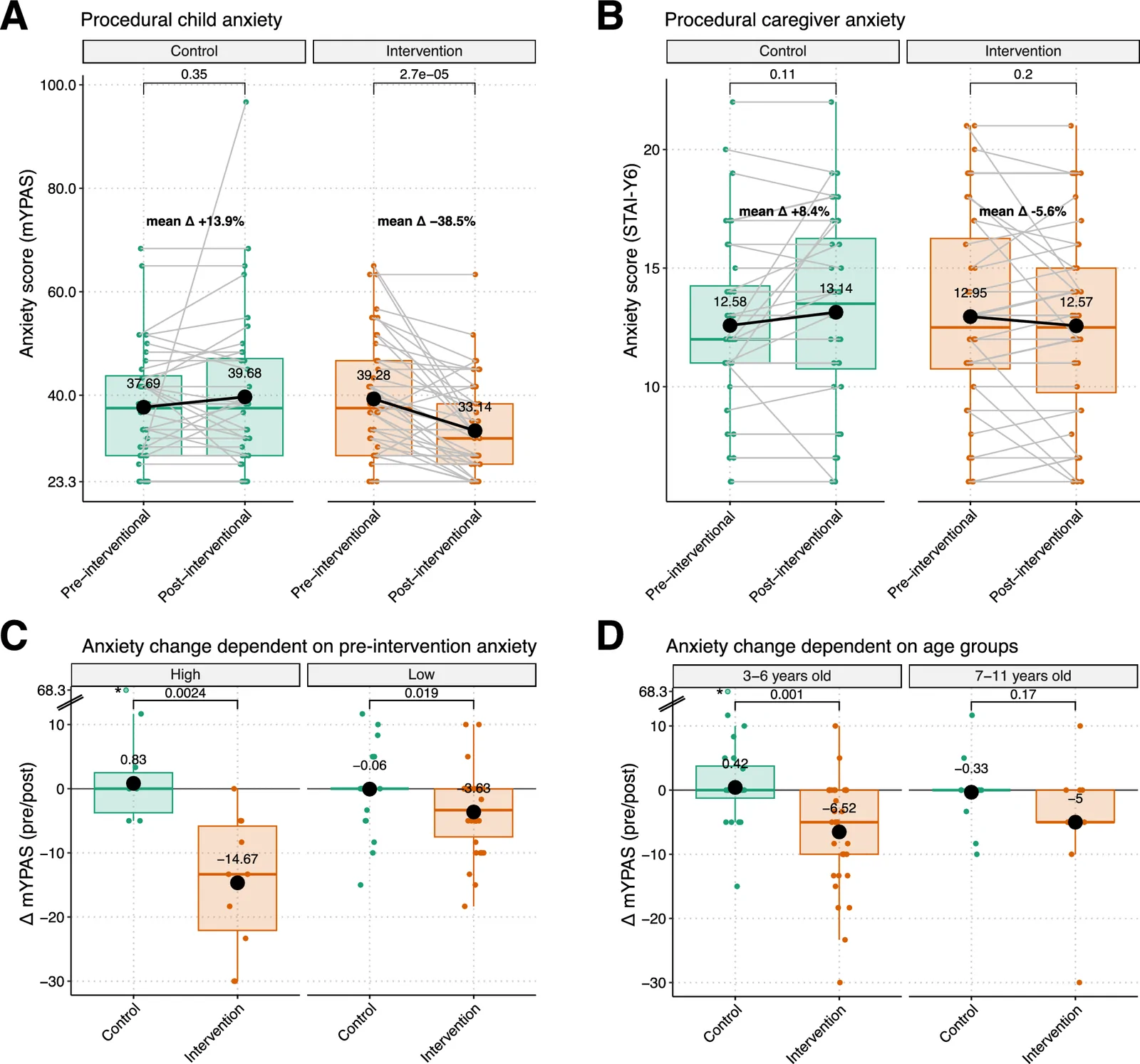
Procedural anxiety. Panel (**A**) (child) and (**B**) (caregiver) depict procedural anxiety before and after music intervention or control (n = 36 control and n = 44 intervention child-caregiver pairs). Panel (**C**) depicts the anxiety change in children dependent on high (top 20%; n = 7 control and n = 10 intervention participants) or low (lower 80%; n = 29 control and n = 34 intervention participants) pre-intervention anxiety. Panel (**D**) depicts the anxiety change in children dependent on age group (3–6 years; n = 21 control and n = 33 intervention participants) versus 7–11 years (n = 15 control and n = 11 intervention participants). Boxplots show median, interquartile range, and individual observations. Means are indicated by black dots and corresponding values. P values were determined by one-sided Mann–Whitney U tests.

Figure 3 depicts the preoperative anxiety changes before and after the intervention. While there was a non-significant tendency toward anxiety increase in the control group, the intervention group showed an overall anxiety decrease by 38.5% in mYPAS levels (p < 0.00001) (Fig. 3A), corresponding to a standardized mean difference (SMD) of -0.8 (95% CI -1.22 to -0.31). The five study participants with low self-reported usage intensity showed no anxiolytic effect. Anxiety changes in adults showed the same general trends, but were only minor and statistically insignificant (Fig. 3B). The corresponding effect size was small to moderate (SMD -0.47, 95% CI -0.92 to -0.03).

Following the hypothesis that patients with particularly high baseline anxiety might benefit most from the music intervention, we performed a subgroup analysis by stratifying the cohort into the top 20% versus the lower 80% based on pre-intervention anxiety scores. Of the 17 participants in the high anxiety group, 15 had baseline anxiety scores ≥ 1 standard deviation above the mean, while 2 were just below this threshold. A significantly greater reduction in anxiety was observed in the high-anxiety group compared to the low-anxiety group (− 14.6 vs. − 3.6 points on the mYPAS scale) (Fig. 3C). Additionally, age-based stratification showed that younger children (3–6 years) were more likely to benefit from the music intervention compared to older children (7–11 years) (Fig. 3D). The anxiolytic effect was particularly pronounced in children aged 3–6 years (SMD -0.9, 95% CI -1.45 to -0.30) and in those with high baseline anxiety (SMD -1.7, 95% CI -2.84 to -0.51). Notably, no significant correlation was found between age and pre-interventional mYPAS scores (Pearson r = 0.06, p = 0.61), indicating that high baseline anxiety and younger age independently contribute to an increased benefit from the music intervention. Age-adjusted sensitivity analyses using a linear mixed-effects model with time, group, age category (3–6 vs. 7–11 years), and all interaction terms confirmed the primary findings. Age did not significantly influence treatment response, whereas the time-by-group interaction remained significant after model selection, indicating that the intervention effect was robust to baseline age differences.

### The effect of MuMa on secondary endpoints

**Fig. 4:**
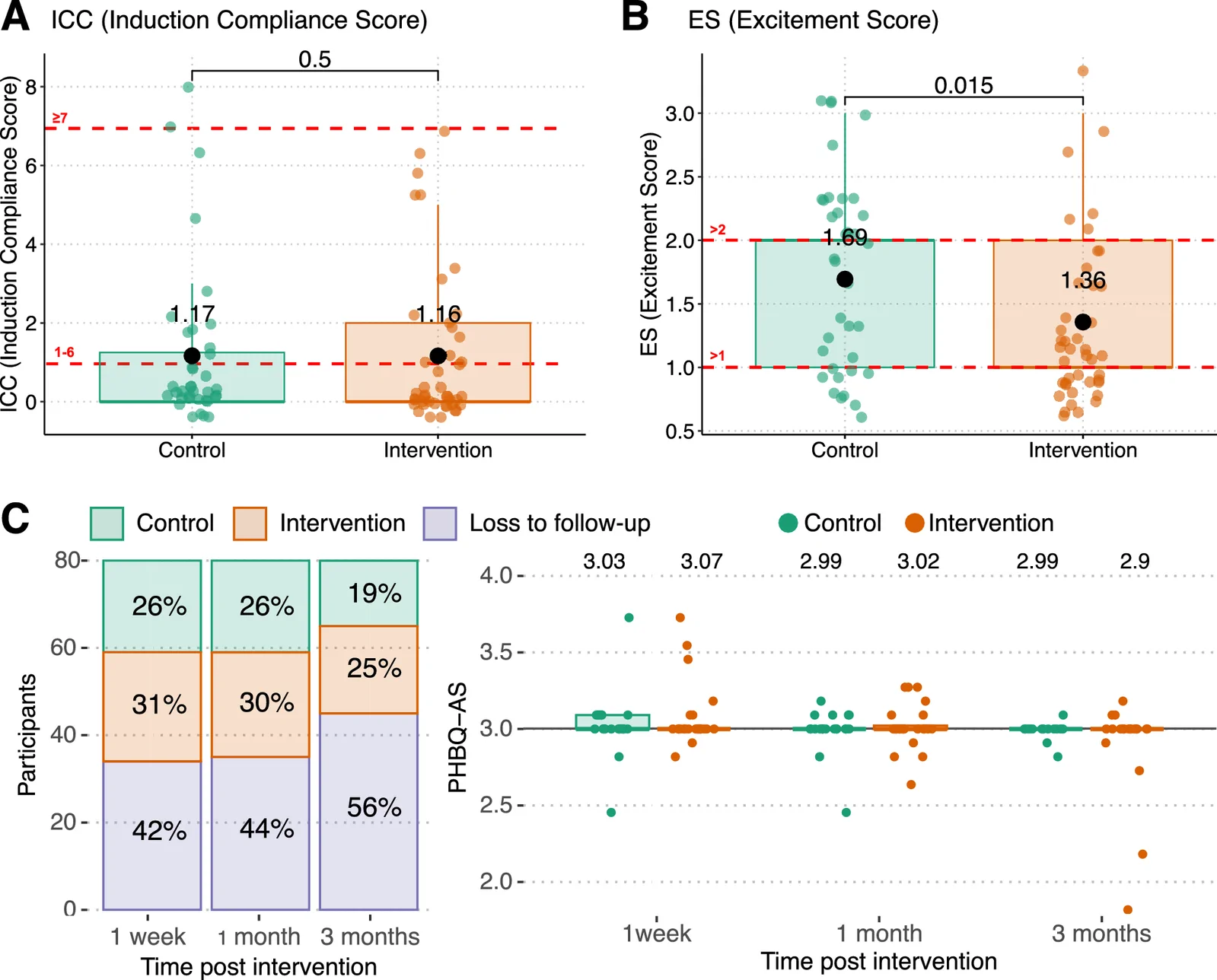
Secondary outcome measures. Panel (**A**) depicts the ICC (Induction Compliance Checklist) assessed at the induction of anesthesia after completion of the music intervention (n = 36 control and n = 43 intervention participants). Red dashed lines indicate segments of excellent < 1, moderate (1–6) and poor (7–10) compliance^28^. Panel (**B**) depicts the ES (Excitement Score), assessed upon arrival in the wake-up area after completion of the surgical procedure (n = 36 control and n = 42 intervention participants). Red dashed lines indicate segments of none (1), marked (2) and substantial (3) postanesthetic agitation^29^. Panel (**C**) depicts the PHBQ-AS (Post-Hospitalization Behavioral Questionnaire – Ambulatory Surgery) assessed one week, 1 month and 3 months after surgery/music intervention. Values > 3 indicate maladaptive behavior, while values < 3 reflect positive behavioral changes^31^. Boxplots show median, interquartile range, and individual observations. Means are indicated by black dots and corresponding values (Panels A and B). P values were determined by one-sided Mann–Whitney U tests.

No substantial differences were observed in the secondary endpoints between the intervention and control groups. Over 70% of participants in both groups exhibited excellent compliance during anesthesia induction, with no significant variation in ICC levels between the groups (Fig. 4A). Only 2 patients in the control group (5.6%) and 1 patient in the intervention group (2.4%) exhibited poor compliance during induction. Postoperative agitation, assessed in the recovery room, showed a small but statistically significant difference in agitation levels between the two groups (p = 0.015), with the intervention group exhibiting slightly lower ES levels (1.36 vs. 1.69) (Fig. 4B). The proportion of substantial and marked agitation was moderately lower in the intervention group compared to the control group (7.1% vs. 13.9%, and 21.4% vs. 41.7%, respectively). None of the participants in either group exhibited significant maladaptive postoperative behavioral changes over the course of the three-month postoperative follow-up (Fig. 4C). HR and HRV were assessed as physiological markers of anxiety. Analyses of the available recordings revealed a main effect of time segment on HR (p = 0.049) and HRV (p = 0.008), but no significant group effect or group-by-time interaction. HR increased and HRV decreased over time irrespective of group allocation, indicating increasing physiological arousal closer to surgery (Suppl. Figure 4).

## Discussion

While previous pediatric music intervention studies often used single-song designs^12–19,39^, this study is, to our knowledge, the first to implement a comprehensive musicological approach via an interactive tablet/smartphone app, which integrated well into clinical practice and was well-received by participants. This randomized controlled trial supports the hypothesis that tablet app-based delivery of an interactive music intervention can reduce preoperative anxiety in children to a clinically relevant extent.

The observed treatment effect was large according to established effect size conventions^35^ (SMD ≈ -0.8), indicating that the reduction in anxiety was not only statistically significant but also clinically meaningful. Particularly pronounced benefits were observed among younger children and those with high baseline anxiety, for whom effect sizes were even larger, suggesting that children with greater emotional vulnerability may derive the greatest benefit from this intervention. In contrast, parental anxiety was only modestly affected.

Analyses of physiological data suggest that HR increased and HRV decreased in closer proximity to surgery, independent of the intervention. While compliance during anesthesia induction (ICC) was excellent in both groups, agitation in the recovery room (ES) was modestly, but statistically significantly reduced in the intervention group. No relevant postoperative maladaptive behavioral changes were observed in either group. The magnitude of the observed treatment effect is comparable to, or exceeds, that reported for other non-pharmacological anxiolytic interventions in children, including music-based and virtual reality approaches^12–19,39,40^. Given the presence of several parallel distraction strategies in our high-resource setting, the observed effect size likely represents a conservative estimate of the intervention’s true impact, which may be greater in lower-resource or high-volume clinical environments.

An inherent drawback of clinical trials for overt non-pharmacological interventions is the inevitable lack of blinding^41^. Accordingly, this study represents an open-label randomized controlled trial, as neither participants nor investigators were blinded. The absence of assessor blinding introduces a risk of detection bias, particularly for observer-rated endpoints such as mYPAS anxiety scores. A further limitation is the retrospective registration of the trial. Although the study protocol, eligibility criteria, primary and secondary outcomes, and statistical analysis plan were defined before data collection and remained unchanged throughout the study, retrospective registration may increase the perceived risk of selective reporting bias. Furthermore, the monocentric design, conducted at a tertiary university children’s hospital, limits the generalizability of our findings to other clinical and healthcare settings. An additional limitation is the baseline age imbalance between groups, with younger children overrepresented in the intervention group. As younger age was associated with greater treatment benefit, this imbalance could have influenced the observed effect size. Nevertheless, age-adjusted sensitivity analyses confirmed the robustness of the primary findings. To minimize inter-observer bias, all primary endpoints were assessed by a single investigator; however, this may have increased susceptibility to systematic bias. Given the predominantly non-chronic nature of the patient cohort, future research should explore whether similar anxiolytic effects can also be achieved in patients with chronic conditions or under repeated procedures. Although the initial target of 100 patients per group was not met due to COVID-19–related logistical constraints, the sample size was sufficiently powered for the primary endpoint and allowed age-based stratification as well as subgroup analyses of highly anxious patients. Moreover, compared with other studies on procedural anxiety reduction in children, the sample size was relatively large. A further limitation is the substantial loss to follow-up for PHBQ-AS assessments, particularly at later time points. While attrition is common in longitudinal post-discharge follow-up studies and was comparable between study groups, the resulting reduction in sample size may have limited the ability to detect subtle differences in postoperative behavioral outcomes.

A key strength of the study is its naturalistic design, evaluating the intervention under routine clinical conditions in the presence of various alternative anxiolytic strategies (e.g., handheld devices, books, games). This design also included the deliberate retention of anxiolytic premedication with midazolam; however, the primary outcome of child anxiety was assessed before midazolam administration and was therefore not influenced by pharmacological anxiolysis. In contrast, standard preoperative midazolam may have attenuated intervention effects on secondary outcomes assessed thereafter, particularly ICC and ES, potentially masking additional benefits of the music intervention. The naturalistic setting, while ecologically valid, impaired HR/HRV data quality, allowing reliable analysis only in a subset of patients. Moreover, more stringent control of intervention time and engagement would have permitted more definitive conclusions regarding changes in autonomic arousal. Nevertheless, secondary endpoints such as arousal in the post-anesthesia care unit – measured after midazolam – still demonstrated favorable effects in the intervention group. Parental anxiety was also considered, given the well documented transference and counter transference effects in pediatric procedural anxiety^42^. However, no significant associations between caregiver and child anxiety were observed.

The main strength of this study is its sophisticated intervention design, grounded in music therapeutic principles, targeting child-friendly coping strategies and validation of emotions. The pre-selection of 3 × 15 songs, presented through age-appropriate graphical representations across three graphical interfaces, allows children to select songs of their liking and at their own speed, a feature previously linked to positive effects in adults^44^. Additionally, the intervention’s adaptability to various healthcare settings and compatibility with personal tablets or smartphones could enhance its future applicability in routine practice. As a low-resource, easily deployable digital intervention, the app has potential relevance for perioperative care in both high- and low-resource environments. Future research should consider expanding the song selection to include English-language tracks and explore the applicability of the anxiolytic concept in additional healthcare settings. Integrating real-time digital usage analytics may enhance personalization and support culturally adaptable versions of the intervention.

## Conclusions

In conclusion, this randomized clinical trial showed a statistically significant and clinically meaningful reduction of procedural anxiety through a tablet app-based music intervention specifically developed for pediatric perioperative care. The intervention was well accepted and readily integrated into clinical practice, requiring minimal resources and no specialized training, with considerable potential for adaptation to other healthcare settings and patient populations. Further multicenter evaluations should assess its scalability and performance across diverse clinical environments.

## Declaration

## Supplementary Information

Supplementary Information 1.

Supplementary Information 2.

Supplementary Information 3.

## Data Availability

The data underlying all analyses and figures presented in this study are provided in Supplementary_Data_1.xlsx. All data have been de-identified and are shared in accordance with applicable ethical and data protection requirements.

## Abbreviations

CI: Confidence interval
CONSORT: Consolidated standards of reporting trials
COVID-19: Coronavirus disease 2019
CV: Coefficient of variation
EcgMove3: HRV sensor used for heart rate variability measurements
ES: Excitement score
HR: Heart rate
HRV: Heart rate variability
ICC: Induction compliance checklist
MAC: Minimum alveolar concentration
MuMa: Acronym for interactive music intervention app (“Music match”)
mYPAS: Modified yale preoperative anxiety scale
PHBQ-AS: Post-hospitalization behavioral questionnaire for ambulatory surgery
RCT: Randomized controlled trial
SD: Standard deviation
SMD: Standardized mean difference
STAI-Y6: State-trait anxiety inventory short form Y6
STAI-YT: State-trait anxiety inventory version YT
UTN: Universal trial number

## Code availability

Custom R scripts used for data processing, statistical analyses, and figure generation are available from the corresponding author upon reasonable request.
