# Supplementary material for: Effectiveness of an interactive music tablet intervention to reduce procedural anxiety in children: a randomized controlled trial
Source: Sci Rep. 2026 Aug 24;16:26483. doi: 10.1038/s41598-026-66888-1 (PMC13503897; doi:10.1038/s41598-026-66888-1)
Supplement: Supplementary file 1 [file 41598_2026_66888_MOESM2_ESM.pdf]

Figure 1: CONSORT 2025 Flow Diagram

Flow diagram of the progress through the phases of a randomised trial of two groups (that is, enrolment, intervention allocation, follow-up, and data analysis)

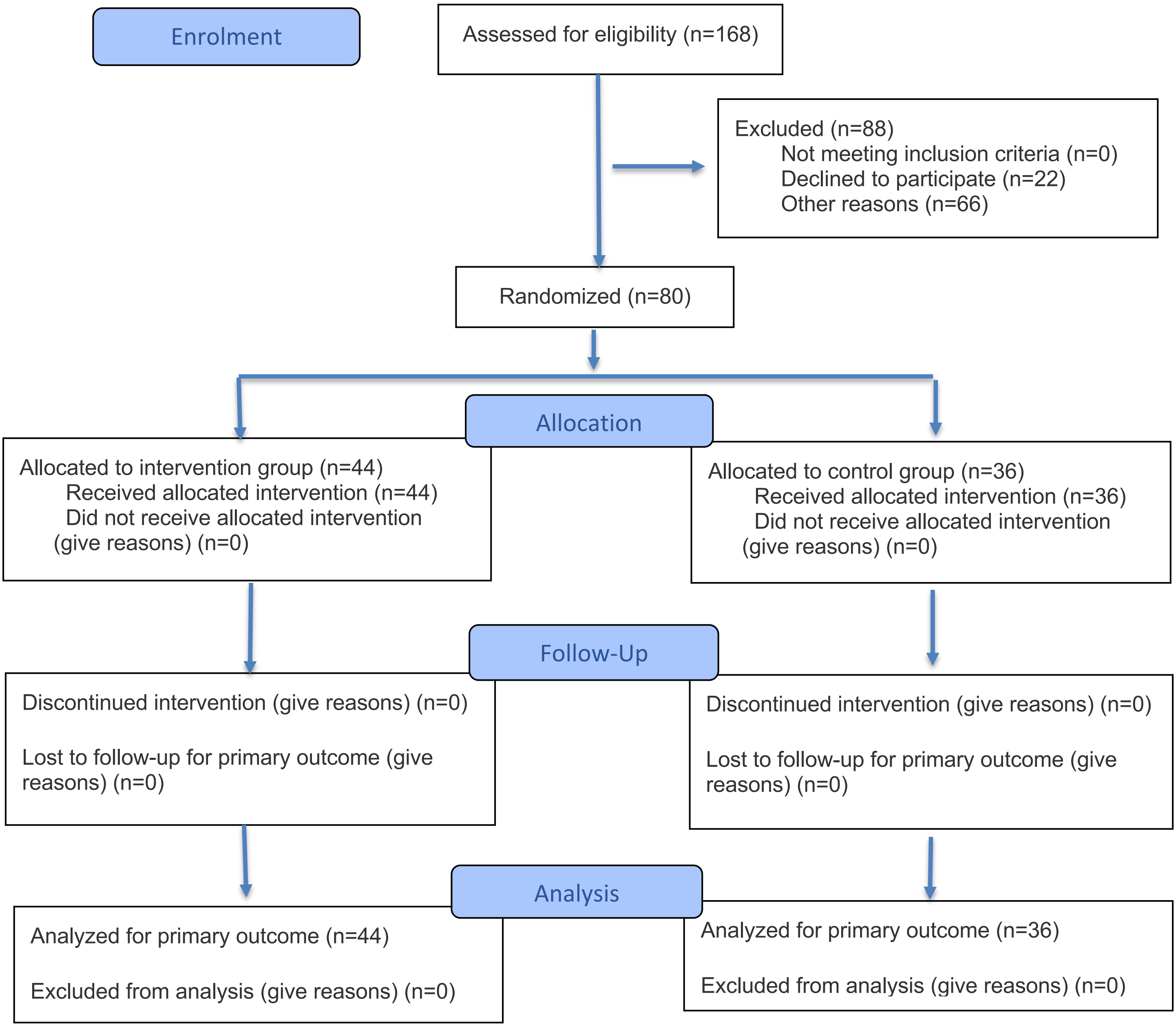

Citation: Hopewell S, Chan AW, Collins GS, Hróbjartsson A, Moher D, Schulz KF, et al. CONSORT 2025 Statement: updated guideline for reporting randomised trials. BMJ. 2025; 388:e081123. https://dx.doi.org/10.1136/bmj-2024-081123
© 2025 Hopewell et al. This is an Open Access article distributed under the terms of the Creative Commons Attribution License (https://creativecommons.org/licenses/by/4.0/), which permits unrestricted use, distribution, and reproduction in any medium, provided the original work is properly cited.

CONSORT 2025 checklist of information to include when reporting a randomised trial*

Section / Topic	No	CONSORT 2025 checklist item description	Reported on page no.
**Title and abstract**			
Title and structured abstract	1a	Identification as a randomised trial	1/line2
	1b	Structured summary of the trial design, methods, results, and conclusions	3/lines 64-96
**Open science**			
Trial registration	2	Name of trial registry, identifying number (with URL) and date of registration	6-7/lines 163-165
Protocol and statistical analysis plan	3	Where the trial protocol and statistical analysis plan can be accessed	6/lines 165-166
Data sharing	4	Where and how the individual de-identified participant data (including data dictionary), statistical code and any other materials can be accessed	23/lines 539-542
Funding and conflicts of interest	5a	Sources of funding and other support (e.g., supply of drugs), and role of funders in the design, conduct, analysis and reporting of the trial	26/lines 682-686
	5b	Financial and other conflicts of interest of the manuscript authors	26/lines 710-711
**Introduction**			
Background and rationale	6	Scientific background and rationale	5-6/lines 11-140
Objectives	7	Specific objectives related to benefits and harms	6/lines 141-150
**Methods**			
Patient and public involvement	8	Details of patient or public involvement in the design, conduct and reporting of the trial	7/lines 176-180
Trial design	9	Description of trial design including type of trial (e.g., parallel group, crossover), allocation ratio, and framework (e.g., superiority, equivalence, non-inferiority, exploratory)	6/lines 153-156
Changes to trial protocol	10	Important changes to the trial after it commenced including any outcomes or analyses that were not prespecified, with reason	7/lines 168-172
Trial setting	11	Settings (e.g., community, hospital) and locations (e.g., countries, sites) where the trial was conducted	6/lines 153-156

Eligibility criteria	12a	Eligibility criteria for participants	7/lines 182-185
	12b	If applicable, eligibility criteria for sites and for individuals delivering the interventions (e.g., surgeons, physiotherapists)	Not applicable
Intervention and comparator	13	Intervention and comparator with sufficient details to allow replication. If relevant, where additional materials describing the intervention and comparator (e.g., intervention manual) can be accessed	8-9/lines 200-224
Outcomes	14	Pre-specified primary and secondary outcomes, including the specific measurement variable (e.g., systolic blood pressure), analysis metric (e.g., change from baseline, final value, time to event), method of aggregation (e.g., median, proportion), and time point for each outcome	9-10/lines 225-253
Harms	15	How harms were defined and assessed (e.g., systematically, non-systematically)	10/lines 245-252
Sample size	16a	How sample size was determined, including all assumptions supporting the sample size calculation	14/lines 354-363
	16b	Explanation of any interim analyses and stopping guidelines	14/lines 361-363
Randomisation:			
Sequence generation	17a	Who generated the random allocation sequence and the method used	7/lines 182-184
	17b	Type of randomisation and details of any restriction (e.g., stratification, blocking and block size)	7/lines 184-185
Allocation concealment mechanism	18	Mechanism used to implement the random allocation sequence (e.g., central computer/telephone; sequentially numbered, opaque, sealed containers), describing any steps to conceal the sequence until interventions were assigned	7-8/lines 185-190
Implementation	19	Whether the personnel who enrolled and those who assigned participants to the interventions had access to the random allocation sequence	7-8/188-190
Blinding	20a	Who was blinded after assignment to interventions (e.g., participants, care providers, outcome assessors, data analysts)	8/lines 191-196
	20b	If blinded, how blinding was achieved and description of the similarity of interventions	Not applicable
Statistical methods	21a	Statistical methods used to compare groups for primary and secondary outcomes, including harms	13/lines 320-332

	21b	Definition of who is included in each analysis (e.g., all randomised participants), and in which group	13/lines 340-343
	21c	How missing data were handled in the analysis	13-14/lines 343-344
	21d	Methods for any additional analyses (e.g., subgroup and sensitivity analyses), distinguishing prespecified from post-hoc	13/lines 332-341
**Results**			
Participant flow, including flow diagram	22a	For each group, the numbers of participants who were randomly assigned, received intended intervention, and were analysed for the primary outcome	14/lines 358-361 (Figure 2)
	22b	For each group, losses and exclusions after randomisation, together with reasons	14/lines 358-366 (Figure 2)
Recruitment	23a	Dates defining the periods of recruitment and follow-up for outcomes of benefits and harms	6/lines 158-163
	23b	If relevant, why the trial ended or was stopped	Not applicable
Intervention and comparator delivery	24a	Intervention and comparator as they were actually administered (e.g., where appropriate, who delivered the intervention/comparator, how participants adhered, whether they were delivered as intended [fidelity])	8-9/lines 200-222
	24b	Concomitant care received during the trial for each group	8/lines 201-206
Baseline data	25	A table showing baseline demographic and clinical characteristics for each group	15/377-378 (Table 1)
Numbers analysed, outcomes and estimation	26	For each primary and secondary outcome, by group:<br>● the number of participants included in the analysis<br>● the number of participants with available data at the outcome time point<br>● result for each group, and the estimated effect size and its precision (such as 95% confidence interval)<br>● for binary outcomes, presentation of both absolute and relative effect size	15/lines 365-373 (numbers analyzed and missing data); 16-17/lines 402-409, lines 419-

			430 (Figure 3 – main endpoint child anxiety mYPAS, caregiver anxiety STAI); 17-18/lines 430-442 (Figure 4 – secondary endpoints ICC, ES, PHBQ-AS); 18/lines 443-447 (Suppl. Fig. 4 – secondary endpoint HRV);
Harms	27	All harms or unintended events in each group	16/lines 393-394
Ancillary analyses	28	Any other analyses performed, including subgroup and sensitivity analyses, distinguishing pre-specified from post-hoc	17/lines 410-424 (Figure 3C-D; pre-specified subgroup analyses); 17/lines 424-429 (post hoc age-adjusted

			sensitivity analysis).
**Discussion**			
Interpretation	29	Interpretation consistent with results, balancing benefits and harms, and considering other relevant evidence	18/lines 449-472
Limitations	30	Trial limitations, addressing sources of potential bias, imprecision, generalisability, and, if relevant, multiplicity of analyses	19-21/lines 473-530

*We strongly recommend reading this statement in conjunction with the CONSORT 2025 Explanation and Elaboration and/or the CONSORT 2025 Expanded Checklist for important clarifications on all the items. We also recommend reading relevant CONSORT extensions. See www.consort-spirit.org.

Citation: Hopewell S, Chan AW, Collins GS, Hróbjartsson A, Moher D, Schulz KF, et al. CONSORT 2025 Statement: updated guideline for reporting randomised trials. BMJ. 2025; 388:e081123. https://dx.doi.org/10.1136/bmj-2024-081123.

© 2025 Hopewell et al. This is an Open Access article distributed under the terms of the Creative Commons Attribution License (https://creativecommons.org/licenses/by/4.0/), which permits unrestricted use, distribution, and reproduction in any medium, provided the original work is properly cited.
