# Supplementary material for: Effectiveness of an interactive music tablet intervention to reduce procedural anxiety in children: a randomized controlled trial
Source: Sci Rep. 2026 Aug 24;16:26483. doi: 10.1038/s41598-026-66888-1 (PMC13503897; doi:10.1038/s41598-026-66888-1)
Supplement: Supplementary file 2 [file 41598_2026_66888_MOESM1_ESM.docx]

[S1) SUPPLEMENTAL MATERIAL AND METHODS	2](#_Toc232002962)

[Intervention (detailed version)	2](#_Toc232002963)

[Outcomes (detailed version)	3](#_Toc232002964)

[ECG data processing and HRV analysis (detailed version)	4](#_Toc232002965)

[S2) SONG SELECTION OF THE MUSIC INTERVENTION	5](#_Toc232002966)

[**Category A: Strength/Courage**	5](#_Toc232002967)

[**Category B: Fun/Distraction**	6](#_Toc232002968)

[**Category C: Safety/Comfort**	7](#_Toc232002969)

[S3) SUPPLEMENTAL REFERENCES	8](#_Toc232002970)

[S4) SUPPLEMENTAL FIGURE LEGENDS	9](#_Toc232002971)

[**Suppl. fig. 1. Study design.**	9](#_Toc232002972)

[**Suppl. fig. 2. Baseline anxiety and intervention time.**	9](#_Toc232002973)

[**Suppl. fig. 3. Integrated data overview.**	9](#_Toc232002974)

[**Suppl. fig. 4. HRV analysis.**	9](#_Toc232002975)

# S1) SUPPLEMENTAL MATERIAL AND METHODS

## Intervention (detailed version)

Subjects were randomized into one control and one intervention group on the day of the medical procedure. Both groups received the standard of care including a premedication with orally administered benzodiazepine (midazolam) after the music intervention before narcosis induction in the majority of cases, a child-friendly environment and presence of the caregiver until start of narcosis induction. Parents and children were free to apply any additional anxiolytic strategies of their choice in both groups.

While the control group received only standard of care, the intervention group was additionally provided with a 10.1-inch Samsung Galaxy Tablet A T515, preloaded with an anxiolytic application called MuMa, starting in the preoperative setting. The intervention lasted at least 10 minutes. MuMa was developed in an interdisciplinary team of music therapists, psychologists, pediatricians, pediatric surgeons, and nurses and was designed to foster child-caregiver interactions by offering the choice between three main coping strategies to alleviate anxiety: (i) Fun & distraction, (ii) Safety & comfort, and (iii) Courage & strength, each represented by individually designed cartoon characters (**Figure 1 of the main manuscript**). Each icon is linked to a unique graphical interface featuring 15 slightly animated figures, each representing a child-friendly song tailored to the respective anxiety reduction strategy. Within each category, two songs were selected to address the emotions of fear and anger, intended to help children connect with and validate these feelings. Additionally, each category includes two participatory songs that encourage movement, such as clapping or gesturing. The nature of each song is visually represented by its corresponding icon: for example, the angry crocodile in interface (ii) symbolizes an "anger song”, while the dancing octopus in interface (i) represents a "participation song." Children can easily switch back and forth between coping strategies and songs or repeat individual tracks for extended periods of time.

All songs were carefully selected by qualified and experienced music therapists, considering both musical and lyrical elements. Special attention was given to the following aspects: structure (e.g., continuity, repetition or contrast), dynamics (soft, loud or varying), tonality and harmony (major or minor, complex or simple), melody (smooth or jagged), articulation (legato or staccato), rhythm (simple, complex, binary, ternary), tempo (fast or languid, varying or constant), vocal timbre, and narrative content (appropriate for children and coping strategy). Headphones were deliberately omitted to encourage shared listening. The volume could be freely adjusted from 0 to 80 decibels. Safety of devices was tested by the medical technical department and tablets were disinfected before and after use. The anxiolytic concept was designed to enable easy transferability to other hospitals and pediatric healthcare settings, with the option for free download to personal tablets and smartphones in the future.

## Outcomes (detailed version)

Primary outcomes were state anxiety before and after music or control intervention in children and caregivers assessed by standard observational (children) and self-reported (adults) outcome measures. In children, the modified Yale Preoperative Anxiety Scale (**mYPAS**) was used, widely regarded as the gold standard for assessing preoperative anxiety in the 2 to 12-year age group^1,2^. It employs a Likert-scale-based assessment of 27 items across five domains of anxiety-related behaviors: activity, vocalizations, emotional expressivity, arousal, and interaction with parents. This results in a total score ranging from 23.33 to 100. Scores above 30 indicate anxiety^3^, while scores more than one standard deviation above the mean are considered indicative of high anxiety intensity^4^.

For caregivers, the **STAI-Y6** (State-Trait Anxiety Inventory – Short Form Y6) scale was used, which generates Likert-scale-based scores ranging from a total 6 to 24 to reliably measure state anxiety in adults^5,6^. Scores between 11 and 13 indicate moderate anxiety, while scores above 13 reflect high state anxiety^7^.

In addition, general fearfulness of participants was determined on the day of the informed consent (2 days to 4 weeks before surgery). mYPAS was used for children, while STAI-YT was used for adults. The **STAI-YT** (State-Trait Anxiety Inventory – Version YT) is a widely used self-reported Likert-scale-based trait anxiety measure with high reliability across diverse populations^8,9^. Total scores of 20-39 correspond to low anxiety, scores of 40-59 to moderate anxiety and scores of 60-80 to high anxiety^8^.

Secondary outcomes were child compliance at narcosis induction measured by **ICC** (Induction Compliance Checklist), agitation on arrival in the recovery room after surgery measured by ES (Excitement Score) and the degree of maladaptive secondary changes measured by the post-hospitalization behavioral questionnaire – ambulatory surgery (**PHBQ-AS**), assessed one week, one month and 3 months post intervention. The ICC is a validated observational scale with ten items, each scored one point. Scores of 0 indicate optimal compliance, 1-6 moderate compliance, and 7-10 poor compliance during anesthesia induction^10,11^. The **ES** is a three point observational scoring system assessed upon arrival in the recovery room, differentiating between none, marked and substantial postanesthetic agitation^12^. The PHBQ-AS is a questionnaire for parents regarding behavioral changes in their children after discharge^13,14^. The 11 items are rated on a 1-5 Likert scale, with the total score divided by 11 to yield an average score ranging from 1 to 5. A score of 3 indicates no change in behavior post-surgery, while lower scores reflect improved behavior and higher scores indicate maladaptive behavior^14^. All assessments of observational scores were conducted by the same investigator to avoid inter-observer bias.

## ECG data processing and HRV analysis (detailed version)

Electrocardiographic (ECG) recordings were obtained continuously as a somatic indicator of anxiety in children at a sampling frequency of 1024 Hz throughout all procedures. Raw ECG data were first screened using the UnisensViewer (Movisens GmbH; Karlsruhe, Germany). Raw data were processed using the Kubios HRV Premium software (Version 3.0)^15^. Incorrectly or unidentified R-peaks were corrected manually. Artifacts or extended periods of signal distortion led to missing R-peaks, resulting in disproportionately large inter-beat intervals (IBIs). These periods were automatically excluded during further processing based on the criterion of physiologically implausible IBIs (IBI values greater than 1500 ms or less than 300 ms). Corrected IBI time series were exported for later segmentation and analyses in STATA SE 19. Extreme data points for IBI, falling outside the range of two inter-quartile ranges, were excluded from further analyses. RMSSD (square root of the mean of the sum of all squared differences between adjacent RR intervals in milliseconds), as measure of vagally-mediated HRV, and the mean HR were derived from two segment for main analyses and exported respectively. The two segments were defined as the first 5 minutes of the intervention period and the 5 minutes before the median end of the intervention period (to account for differences in length of the intervention period).

# S2) SONG SELECTION OF THE MUSIC INTERVENTION

**Category A: Strength/Courage** (Graphically represented by a tiger)

| **ID:** | **Song title (German)** | **Song title (English translation)** | **Graphical representation** |
| --- | --- | --- | --- |
| A1 | Du hast Angst | You Are Scared | Laughing zebra |
| A2 | Das Wutlied (Rummelplatz) | The Anger Song (Amusement Park) | Cartoon Buffalo |
| A3 | Wo ist die Wut, wenn ich wütend bin | Where is the Anger When I'm Angry | Angry crocodile |
| A4 | Bald geht's mir wieder gut | I'll Be Fine Soon | Hopeful parrot |
| A5 | Pippi Langstrumpf | Pippi Longstocking | Pippi Langstrumpf |
| A6 | Kennst du das auch | Do You Know This Too | Chameleon |
| A7 | Kindermutmachlied | Children's Cheer-Up Song | 2 snugging horses |
| A8 | Tschakka, du schaffst es | You Can Do It | Strong hippopotamus |
| A9 | Ich bin groß | I Am Big | Big elephant |
| A10 | Starke Kinder | Strong Children | Enthusiastic child |
| A11 | Ich bin Tabaluga | I Am Tabaluga | Dragonbaby |
| A12 | Ich wär so gern wie du | I Wish I Were Like You | Monkey |
| A13 | Auf dem Katzentatzentanzfest | At the Kitten Dance Party | Dancing tiger baby |
| A14 | Der Papagei ein Vogel ist | The Parrot is a Bird | Parrot |
| A15 | Die Maus und der Löwe Grüffelo | The Mouse and the Lion Gruffalo | Lion |

Songs toward additional coping mechanisms in category A:

- 2 songs toward validation and letting go of sadness: A1 + A6
- 2 songs toward validation and letting go of anger: A2 + A3
- 2 songs toward participation: A13 + A14

**Category B: Fun/Distraction** (Graphically represented by an octopus)

| **ID:** | **Song title** | **Song title (English translation)** | **Graphical representation** |
| --- | --- | --- | --- |
| B1 | Wie man Geister fängt | How to Catch Ghosts | Ghost |
| B2 | Ich möchte mal wer anders sein | I Want to Be Someone Else | Crab |
| B3 | Ringedingeding | Ringadingading | Octopus |
| B4 | Seeräuber-Opa Fabian | Pirate Grandpa Fabian | Pirat |
| B5 | Ein Tag im Zoo | A Day at the Zoo | Flamingo |
| B6 | Das Froschlied | The Frog Song | Frog |
| B7 | Die Affen rasen durch den Wald (Cicero) | The Monkeys Race Through the Forest (Cicero) | Clownfish |
| B8 | Das Riech-, Keuch-, Nies- Und Schnarch-Lied | The Smell, Pant, Sneeze, and Snore Song | Walrus |
| B9 | Unter dem Meer | Under the Sea | Whale |
| B10 | Hakuna Matata | Hakuna Matata | Seal |
| B11 | Hokus Pokus Fidibus | Hocus Pocus Fidibus | Wizard |
| B12 | Schweinhornschnabelohrwurm | Pig Horn Snout Earworm | Ray |
| B13 | Ein Popel (Gerhrdt Schöne) | A Booger (Gerhardt Schöne) | Turtle |
| B14 | Hexe Pauliquaul | Witch Pauliquaul | Green witch |
| B15 | Manchmal hab ich Wut | Sometimes I Am Angry | Lanternfish with sharp teeth |

Songs toward additional coping mechanisms in category B:

- 2 songs toward validation and letting go of sadness: B1+ B14
- 2 songs toward validation and letting go of anger: B2 + B15
- 2 toward participation: B3 + B11

**Category C: Safety/Comfort** (Graphically represented by a bear)

| **ID:** | **Song title** | **Song title (English translation)** | **Graphical representation** |
| --- | --- | --- | --- |
| C1 | Hab ich mal Angst | Sometimes I'm Scared | Cute mouse |
| C2 | Angst (Lila Lindwurm) | Fear (Purple Lindworm) | Whistling bird |
| C3 | Fauchi der kleine Tiger | Fauchi the Little Tiger | Cute tiger |
| C4 | Row Row Row Your Boat | Row Row Row Your Boat | Frog in a boat |
| C5 | Ich schenk dir einen Regenbogen | I'll Give You a Rainbow | Rainbow |
| C6 | Auf dem Rücken von 'nem Drachen | On the Back of a Dragon | Red dragon |
| C7 | Der Baum des Lebens | The Tree of Life | Tree |
| C8 | Die Eule mit der Beule | The Owl with the Bump | Owl |
| C9 | Macht doch nichts | No Big Deal | Skunk |
| C10 | Wisst ihr, was die Bienen träumen | Do You Know What the Bees Dream | Bee |
| C11 | Probier’s mal mit Gemütlichkeit | The Bare Necessities | Bear |
| C12 | Der Hummer hat Kummer | The Lobster is worried | Red crab |
| C13 | Zwei kleine Wölfe | Two Little Wolves | Wolf |
| C14 | Weg mit dem Grummel, weg mit dem Groll | Away with the Grumble, Away with the Grudge | Bat |
| C15 | Das Kätzchen Grüffelo | The Little Cat Gruffalo | Cat |

Songs toward additional coping mechanisms in category C:

- 2 songs toward validation and letting go of sadness: C1+ C12
- 2 songs toward validation and letting go of anger: C3 + C14
- 2 toward participation: C3 + C13

# S3) SUPPLEMENTAL REFERENCES

1.	Kain, Z. N. *et al.* Measurement tool for preoperative anxiety in young children: The yale preoperative anxiety scale. *Child Neuropsychol.* **1**, 203–210 (1995).

2.	Yun, R. *et al.* Assessing pediatric perioperative affect: A concise review of research and clinically relevant scales. *Paediatr. Anaesth.* **33**, 243–249 (2023).

3.	Stargatt, R. *et al.* A cohort study of the incidence and risk factors for negative behavior changes in children after general anesthesia. *Paediatr. Anaesth.* **16**, 846–59 (2006).

4.	Eijlers, R. *et al.* Predicting Intense Levels of Child Anxiety During Anesthesia Induction at Hospital Arrival. *J. Clin. Psychol. Med. Settings* **28**, 313–322 (2021).

5.	Marteau, T. M. & Bekker, H. The development of a six-item short-form of the state scale of the Spielberger State-Trait Anxiety Inventory (STAI). *Br. J. Clin. Psychol.* **31**, 301–6 (1992).

6.	Tluczek, A., Henriques, J. B. & Brown, R. L. Support for the reliability and validity of a six-item state anxiety scale derived from the State-Trait Anxiety Inventory. *J. Nurs. Meas.* **17**, 19–28 (2009).

7.	Gould, M. K. *et al.* Emotional Distress, Anxiety, and General Health Status in Patients With Newly Identified Small Pulmonary Nodules: Results From the Watch the Spot Trial. *Chest* **164**, 1560–1571 (2023).

8.	Spielberger, C. D., Gorsuch, R. L., Lushene, R., Vagg, P. R., & Jacobs, G. A. Manual for the State-Trait Anxiety Inventory (STAI: Form Y). *Consult. Psychol. Press* 4–26 (1983).

9.	Kain, Z. N., Mayes, L. C., Caldwell-Andrews, A. A., Karas, D. E. & McClain, B. C. Preoperative anxiety, postoperative pain, and behavioral recovery in young children undergoing surgery. *Pediatrics* **118**, 651–8 (2006).

10.	Kain, Z. N., Mayes, L. C., Wang, S. M., Caramico, L. A. & Hofstadter, M. B. Parental presence during induction of anesthesia versus sedative premedication: which intervention is more effective? *Anesthesiology* **89**, 1147–56; discussion 9A-10A (1998).

11.	Vieco-García, A. *et al.* Comparison of different scales for the evaluation of anxiety and compliance with anesthetic induction in children undergoing scheduled major outpatient surgery. *Perioper. Med. (London, England)* **10**, 58 (2021).

12.	Keegan, N. J., Yudkowitz, F. S. & Bodian, C. A. Determination of the reliability of three scoring systems to evaluate children after general anesthesia. *Anaesthesia* **50**, 200–202 (1995).

13.	Vernon, D. T., Schulman, J. L. & Foley, J. M. Changes in children’s behavior after hospitalization. Some dimensions of response and their correlates. *Am. J. Dis. Child.* **111**, 581–93 (1966).

14.	Jenkins, B. N. *et al.* Revisiting a measure of child postoperative recovery: Development of the Post Hospitalization Behavior Questionnaire for Ambulatory Surgery. *Paediatr. Anaesth.* **25**, 738–745 (2015).

15.	Tarvainen, M. P., Niskanen, J.-P., Lipponen, J. A., Ranta-Aho, P. O. & Karjalainen, P. A. Kubios HRV--heart rate variability analysis software. *Comput. Methods Programs Biomed.* **113**, 210–20 (2014).

# S4) SUPPLEMENTAL FIGURE LEGENDS

**Suppl. fig. 1. Study design.** Study endpoints: mYPAS (modified Yale preoperative anxiety scale), STAI (State anxiety inventory), STAI-Y6 (Short version of STAI), ICC (Induction compliance score), ES (Excitement score), PHBQ-AS (Post-hospitalization behavioral questionnaire for ambulatory surgery), HRV (Heart rate variability measurement).

**Suppl. fig. 2. Baseline anxiety and intervention time.** Panel A (child) and B (caregiver) depict the baseline anxiety levels measured at the time of the day of informed consent by mYPAS for state anxiety in children (range: 23.33 – 100.0) and STAI-YT for trait anxiety in caregivers (range: 20.0 – 80.0) respectively. Red dashed lines mark segments of low, present/moderate and high anxiety^3,8^. SD = Standard deviation. Panel C depicts the interventional observation period from pre- to postinterventional anxiety measurement at the day of the intervention. Boxplots with median and individual values. Mean marked with black dot and printed out. P values were determined by two-sided student’s t-tests.

**Suppl. fig. 3. Integrated data overview.** Heatmap visualization of collected primary and secondary endpoints, alongside additional clinical data for pediatric patients (upper panel) and associated caregivers (lower panel). Gray areas indicate non-availability of data points.

**Suppl. fig. 4. HRV analysis.** Panel A depicts the IBI (Inter beat interval) Heart rate variability (HRV) changes in response to intervention and control conditions. Individual trajectories and group means for interbeat interval (IBI) and root mean square of successive differences (RMSSD) are shown for pre- and post-intervention segments (T1+5 minutes and Median−5 minutes). The lower panel depicts correlations between HRV changes (ΔRMSSD) and changes in anxiety (ΔmYPAS pre- vs. post intervention).
